# Bilateral Basal Ganglia Calcification (Fahr's Syndrome) Secondary to Hypoparathyroidism: A Case Series

**DOI:** 10.1002/ccr3.73198

**Published:** 2026-07-19

**Authors:** Ali Gohar, Moeez Ahmad Saeed, Arham Saif, Azka Irfan, Humza Tariq, Ruman Shahzad, Amna Suhail, Haseeb Ahmad, Muhammad Husnain Ahmad

**Affiliations:** ^1^ Department of Medicine Lahore General Hospital Lahore Punjab Pakistan; ^2^ Department of Medicine Punjab Medical College Faisalabad Punjab Pakistan; ^3^ Punjab Medical College Faisalabad Medical University Faisalabad Punjab Pakistan; ^4^ Department of Nuclear Medicine Pakistan Kidney and Liver Institute & Research Center Lahore Punjab Pakistan; ^5^ Department of Internal Medicine S Tentishev Asian Medical Institute Kant Chuy Province Kyrgyzstan

**Keywords:** bilateral basal ganglia calcification, endocrinopathies, Fahr's syndrome, hypoparathyroidism, seizures

## Abstract

Three cases of Fahr's syndrome presented with seizures and extrapyramidal features, all linked to hypoparathyroidism with disrupted calcium‐phosphate levels. CT confirmed basal ganglia calcification. Anticonvulsants, calcium, and vitamin D controlled seizures. Identifying metabolic triggers early—and involving neurology, endocrinology, and psychiatry—changes outcomes.

## Key Clinical Message

Fahr's syndrome can involve multiple systems requiring a multidisciplinary team approach involving neurophysicians, neuropsychiatrists, and endocrinologists for the identification and management. This case series opens opportunities for researchers to investigate the link of autoimmune and endocrine associations with Fahr's syndrome.

## 1 Introduction

Fahr's syndrome, a rare neurodegenerative disorder, was first described in 1930 by a German neurologist, Karl Fahr, in an 81‐year‐old male during autopsy [1, 2]. It is characterized by the bilateral, symmetrical calcification of the basal ganglia and other deep brain structures such as the thalamus and dentate nuclei, typically secondary to an identifiable metabolic or endocrine abnormality such as hypoparathyroidism [3].

Diagnostic criteria for Fahr's syndrome include bilateral calcifications of the basal ganglia or other parts of the brain, neurological dysfunction with or without psychiatric symptoms, onset in the age of 30–40 years, presence of endocrinopathy such as hypoparathyroidism or mitochondrial disorder [4].

It is distinguished from PFBC, a hereditary idiopathic condition, which follows the autosomal dominant or recessive pattern, including bilateral intracranial calcification, neurological dysfunction with or without psychiatric symptoms, positive family history, onset in the age of 40–50 years, absence of biochemical and metabolic abnormalities, and absence of any toxic, traumatic, and infectious causes of intracranial calcifications [2].

The precise cause of calcium deposition in Fahr's syndrome remains unclear. However, chronic metabolic, endocrine, or autoimmune disorders, most commonly hypoparathyroidism and impaired calcium phosphate metabolism, result in calcification [1, 5]. Persistent hypocalcemia and hyperphosphatemia lead to an elevated calcium phosphate product, causing gradual perivascular and parenchymal calcium deposition in the basal ganglia, thalamus, and dentate nuclei. This calcification process typically evolves over years to decades before becoming radiologically evident and clinically symptomatic. Neurological manifestations, such as movement disorders, seizures, cognitive impairment, and psychiatric symptoms, usually appear only after a critical threshold of calcification is reached. Autoimmune etiologies, including autoimmune hypoparathyroidism and systemic lupus erythematosus, may accelerate disease progression through combined metabolic derangement and microvascular injury [5, 6].

Fahr's syndrome is considered extremely rare, although its true prevalence is not well established. Some reports estimate its occurrence in less than 1 case per 1,000,000 population. Incidental intracranial calcifications consistent with this syndrome are found in approximately 0.3%–1.2% of routine brain CT scans, suggesting that the condition may be underrecognized or under‐diagnosed in clinical practice [7, 8].

Symptoms of Fahr's syndrome are diverse and can vary widely among individuals, depending upon the location of brain calcification. Calcification in the basal ganglia leads to movement disorders such as jerky movements, stiffness, tremors, or dystonia, and sometimes seizures due to disturbed neuronal signaling in this area that normally helps to control movement, muscle tone, and coordination through complex motor circuits. Involvement of the dentate nucleus can further affect balance and coordination. Meanwhile, periventricular calcifications affect nearby white‐matter pathways that connect different parts of the brain responsible for thinking, mood, and behavior, which explains cognitive decline, memory loss, difficulties in concentration, depression, or psychiatric symptoms [3, 5, 9].

Non‐contrast CT of the brain, MRI brain, and plain skull radiograph are the imaging modalities used for the diagnosis of intracranial calcifications, but non‐contrast CT of the brain is the mainstay method to localize and check the extent of calcification [5].

Treatment strategies primarily focus on symptomatic relief and addressing any underlying cause. Some evidence suggests that early diagnosis and intervention may halt the progression of calcifications, leading to significant improvements in cognitive and motor functions [5].

This case series aims to describe the clinical presentation, diagnostic evaluation, and therapeutic management of patients with bilateral basal ganglia calcification (Fahr's syndrome) secondary to chronic endocrinopathies.

## 2 Case History and Examination

### 2.1 Case 1

A 29‐year‐old female, known case of hypothyroidism and epilepsy for about 10 years, presented to the medical emergency department with generalized tonic–clonic seizures (GTC) for 1 week. She underwent total thyroidectomy for multinodular goiter 10 years ago. Post‐surgery, she was taking oral thyroxine 100 μg daily. She was also taking sodium valproate 500 mg twice daily initially and switched to levetiracetam 500 mg twice daily after developing a skin rash. She has been taking oral calcium and vitamin D supplements for the past 10 years. She has no history of diabetes, hypertension, psychiatric illness, stroke, trauma, or addiction. There was no history of recent infection or any focal neurological deficit. At presentation, she was vitally stable. On general physical examination, oral thrush, pus‐filled blisters, and scaly and flaky skin patches were noted (Figure 1A–C). Her neurological examination was normal.

**FIGURE 1:**
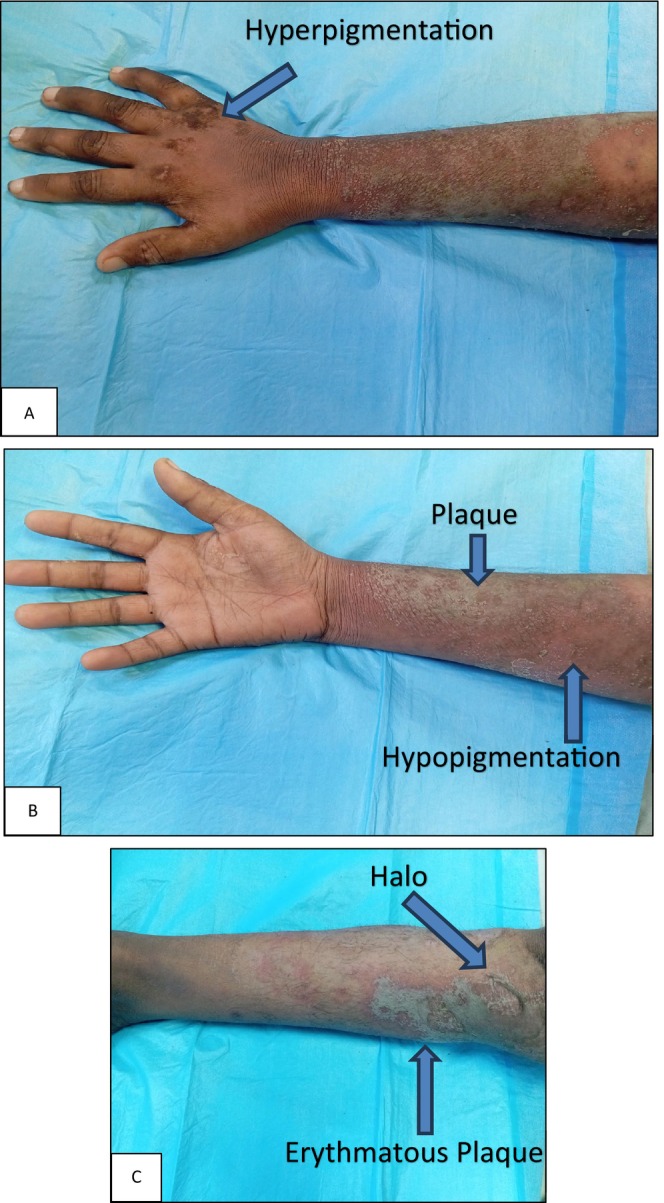
Case 1: (A) Clinical photograph of the dorsum of the hand showing patchy, confluent areas of dark brown hyperpigmentation. The hyperpigmentation is predominantly distributed over the digits and overlying the knuckles (metacarpophalangeal and interphalangeal joints). (B) Clinical photograph of the forearm demonstrating a well‐demarcated, intensely erythematous base covered with thick, elevated plaques. The plaques are bordered by distinct zones of hypopigmentation and multiple clustered, pinpoint pustules distributed across both the dorsal and ventral aspects. (C) Clinical photograph of the pretibial region (shin) highlighting a well‐defined, centralized erythematous plaque. The lesion is characteristically surrounded by a distinct, pale, hypopigmented halo.

### 2.2 Case 2

A 35‐year‐old female presented with on‐and‐off jerky movements of her hands and face for 6 months. The jerky movements were not associated with uprolling of the eyes, urinary and fecal incontinence, tongue biting, frothing from the mouth, or loss of consciousness. She had no fever, trauma, stroke, or addiction. There was a history of depression for 4 months and poor oral intake due to mood changes and oral thrush (Figure 2). She was taking divalproate sodium and carbamazepine. The patient was vitally stable. On examination, positive findings included pallor, oral thrush, and dystrophic nails.

**FIGURE 2:**
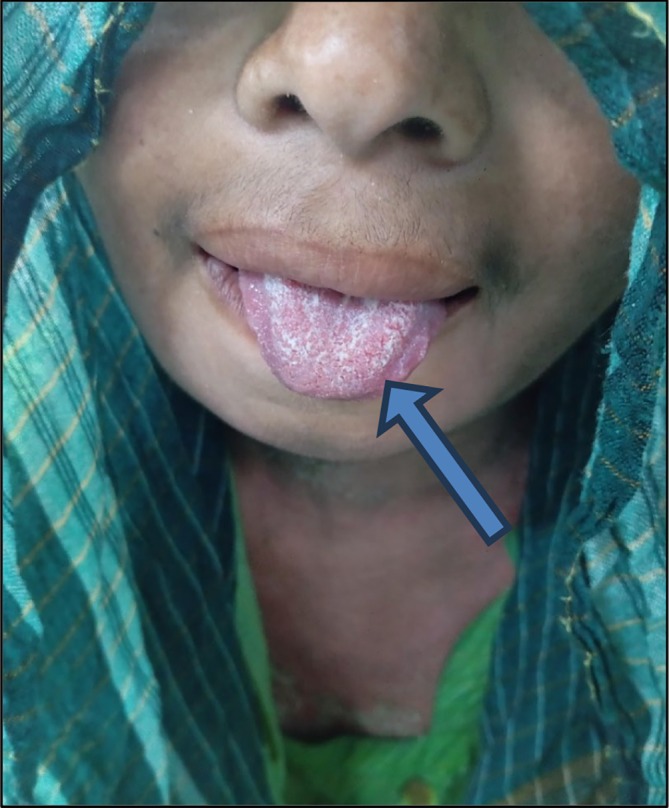
Case 1: Clinical photograph of the oral cavity with tongue protrusion, demonstrating extensive, thick, white pseudomembranous plaques covering the dorsal surface of the tongue, highly characteristic of oral thrush (candidiasis).

### 2.3 Case 3

A 38‐year‐old male patient presented to the emergency department with GTC seizures and altered sensorium for 3 days. His medical history was notable for hypothyroidism, for which he was taking levothyroxine.

## 3 Methods (Differential Diagnosis, Investigations, and Treatment)

### 3.1 Case 1

After history and examination, Fahr's syndrome, vasculitis, and paraneoplastic disease were our differentials. On laboratory investigations, her calcium and phosphate levels were in the normal range (this was attributed to chronic calcium and vitamin D supplementation), whereas her parathormone levels were low. Her hemoglobin was 8.1 g/dL, while the reticulocyte count was 1.3%. The thyroid profile and iron profile were normal. The electroencephalogram (EEG) was normal, as epileptiform discharges may not be captured outside the ictal period. Non‐contrast CT of the brain revealed symmetrical extensive calcification in the bilateral basal ganglia, dentate nuclei, and periventricular regions of the brain (Figure 3A,B). Subsequently, a clinical diagnosis of Fahr's syndrome was made. During her stay, she was treated with intravenous levetiracetam, oral thyroxin, oral prednisolone, betamethasone lotion, and liquid paraffin. Nystatin oral drops, along with calcium and vitamin D supplements, were added to her treatment plan.

**FIGURE 3:**
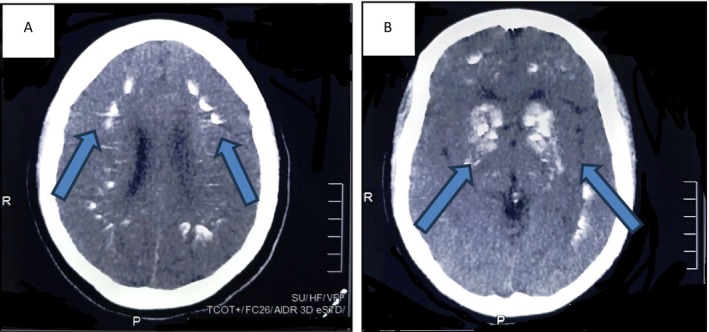
Case 1: (A, B) Non‐contrast axial computed tomography (CT) scan of the brain demonstrating extensive, bilateral, and symmetrical hyperdense calcifications. The calcifications prominently involve the basal ganglia (including the caudate nuclei, putamen, and globus pallidus) and extend significantly into the periventricular white matter regions. Additional punctuate foci of calcification are noted in the frontal subcortical white matter.

### 3.2 Case 2

At the time of presentation, serum calcium and phosphorus were in the normal range. The parathyroid hormone and vitamin D3 levels were low, that is, 5 pg/mL (normal range: 15.1–68.3 pg/mL) and 18 ng/mL (normal: 30–100 ng/mL), respectively. ECG and chest X‐ray were normal. Hemoglobin was 11.1 g/dL while reticulocytes were 1.3%. The thyroid profile was in the normal range. The electroencephalogram (EEG) showed no epileptiform discharge or focal slowing, as such changes might not be captured in the postictal period. Nerve conduction studies were normal. A CT scan of the brain (plain) revealed symmetrical extensive calcification in the bilateral basal ganglia (Figure 4). The stool for occult blood was positive. Colonoscopy revealed internal hemorrhoids. The diagnosis of Fahr's syndrome was made based on the patient's presentation and calcifications seen in her CT brain. In the acute medical unit, the patient was given sodium valproate. She was treated with levetiracetam 1 g, oral prednisolone 10 mg in the morning and 5 mg in the evening, calcium, and vitamin D supplementation were given. Nystatin oral drops were given for oral thrush.

**FIGURE 4:**
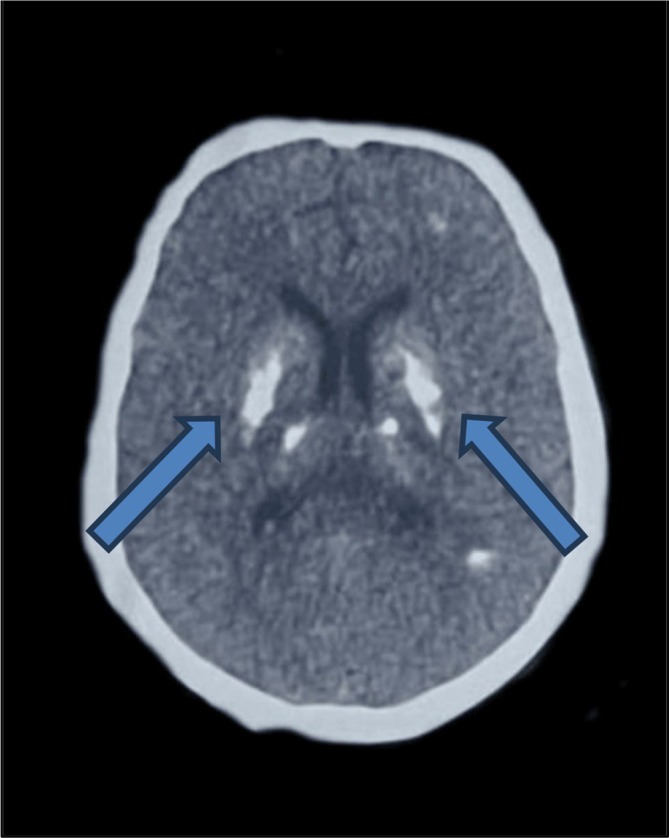
Case 2: Non‐contrast axial computed tomography (CT) scan of the brain revealing dense, well‐defined, bilateral, and symmetrical calcifications restricted primarily to the basal ganglia. Punctate calcified foci are also visible symmetrically within the posterior thalami/periventricular regions, with relative sparing of the overlying cerebral cortex and subcortical white matter.

### 3.3 Case 3

Laboratory investigations revealed low parathyroid hormone levels, while serum calcium and phosphate levels were in the normal range, reflecting ongoing calcium and vitamin D supplementation. Infectious and toxicological screening was negative. A non‐contrast CT scan of the brain showed bilateral basal ganglia calcification. Medication was initiated for seizure control along with calcium and vitamin D supplementation, and calcium and phosphate levels were monitored during the treatment.

## 4 Outcome and Follow Up

### 4.1 Case 1

The patient responded well to the treatment during her 9‐day stay, with no seizure activity observed. The oral thrush improved gradually, and her corrected calcium levels remained at 2.45 mmol/L. She was subsequently discharged. At the 1‐month follow‐up, her biochemical profile was in the normal range with improvement in symptoms.

### 4.2 Case 2

Her condition improved, and she was discharged after 7 days. At 3‐month follow‐up, her oral thrush had improved, and serum calcium, PTH, and vitamin D3 showed improving trends.

### 4.3 Case 3

On discharge, he was asked to have regular follow‐up with a neurologist and endocrinologist to manage symptoms and prevent complications.

Laboratory investigations of all cases are shown in Table 1, and a summary is presented in Table 2. The diagnostic workup for bilateral basal ganglia calcification necessitates a clear distinction between primary (genetic) and secondary (metabolic) causes. In our series, the presence of hypocalcemia and hyperphosphatemia in the context of low PTH levels provided a clear diagnostic rationale for hypoparathyroidism; however, these metabolic findings are characteristic of Fahr's syndrome, we must acknowledge the limitation that genetic testing, the definitive method for diagnosing Primary Familial Brain Calcification (PFBC), was not available at our center.

**TABLE 1:** Laboratory investigations of cases.

Parameters	Case no. 1	Case no. 2	Case no. 3
Parathyroid hormone (PTH) (pg/mL)	4	5	6
Serum calcium (mg/dL)	8.1	8.3	8.2
Serum Manesium (mg/dL)	2.1	2.0	2.3
Serum Phosphorus (mg/dL)	4.1	3.9	3.4
Vitamin D (ng/mL)	22	18	21
ANA	Normal	Normal	Normal
ENA Panel	Normal	Normal	Normal
HbsAg	−ve	−ve	−ve
Anti‐HCV	−ve	−ve	−ve
Anti‐HIV	−ve	−ve	−ve
Toxicology screen	−ve	−ve	−ve

**TABLE 2:** Comparative clinical, biochemical, radiological, and therapeutic characteristics of cases.

Case	Age	Gender	Clinical manifestations	Laboratory findings	Imaging conclusion (CT brain palin)	Treatment
1	29 years	Female	Recurrent GTC seizures; mucocutaneous involvement (oral thrush, pus‐filled blisters, scaly skin); long‐standing hypothyroidism and epilepsy post thyroidectomy	Low PTH; normal calcium and phosphate; anemia (Hb 8.1 g/dL); normal EEG	Extensive symmetrical calcification of bilateral basal ganglia, dentate nuclei, and periventricular regions	IV levetiracetam, oral thyroxine, systemic and topical steroids, nystatin, calcium and vitamin D supplements
2	35 years	Female	Chronic focal jerky movements without loss of consciousness; depression; oral thrush; dystrophic nails	Low PTH (5 pg/mL); low vitamin D3; normal calcium and phosphate; normal EEG and NCS	Symmetrical bilateral basal ganglia calcification	Levetiracetam, sodium valproate, oral prednisolone, calcium and vitamin D, nystatin drops
3	38 years	Male	Acute GTC seizures with altered sensorium; known hypothyroidism	Low PTH; normal calcium and phosphate (on supplementation); negative infectious and toxicology screen	Bilateral basal ganglia calcification	Antiepileptic drugs, calcium and vitamin D supplementation, specialist follow‐up

The differential diagnosis of bilateral basal ganglia calcification includes primary familial brain calcification (Fahr disease), pseudohypoparathyroidism, mitochondrial disorders, congenital or acquired infections (e.g., TORCH infections, HIV), toxic exposures and other inherited metabolic disorders. In our patients, persistently low levels of parathyroid hormone along with clinical evidence of hypoparathyroidism strongly suggested secondary Fahr disease due to hypoparathyroidism. No known family history of primary familial brain calcification, no clinical features of mitochondrial disease or pseudohypoparathyroidism, and infectious and toxicological workups were negative when performed. Genetic testing was unavailable, but the biochemical abnormalities and clinical context strongly favored a secondary metabolic etiology over a primary genetic disorder.

## 5 Discussion

The association between endocrinopathies, mainly hypoparathyroidism, and Fahr's syndrome has been consistently appreciated, with parathyroid hormone (PTH) deficiency believed to impair calcium‐phosphate homeostasis, leading to metastatic calcification in the brain parenchyma [10, 11, 12]. The mechanism involves chronic hypocalcemia and hyperphosphatemia, which reduce calcium solubility and promote its deposition in tissues, including cerebral vasculature and neurons [5]. In our series, all patients showed persistently low PTH levels, confirming underlying hypoparathyroidism, underscoring the strong association between parathyroid dysfunction and Fahr's pathology. Although serum calcium and phosphate levels were within the normal range at the time of presentation in our patients, these findings most likely reflected calcium and vitamin D supplementation initiated before hospital presentation. Intracranial calcification develops because of chronic disturbances in calcium‐phosphate homeostasis over the span of many years rather than isolated biochemical measurements at a single time point. Therefore, normal serum calcium and phosphate at presentation do not exclude longstanding hypoparathyroidism as the underlying cause of intracranial calcification.

Moreover, our findings extend beyond parathyroid involvement, revealing wider endocrine issues, such as hypothyroidism and other autoimmune conditions (e.g., pustular psoriasis), which may point towards an autoimmune polyendocrine syndrome‐like picture. While the exact pathophysiological mechanism remains unclear, immune‐mediated vascular inflammation and blood–brain barrier disruption have been proposed as potential contributors to abnormal calcium deposition [13]. The presence of oral thrush, anemia, and skin manifestations in some cases supports a systemic inflammatory or immunologic component, aligning with reports by Nawaz et al., in which Fahr's syndrome co‐occurred with autoimmune polyendocrine syndrome and neuromyelitis optica spectrum disorder [13].

Although autoimmune hypoparathyroidism and autoimmune polyendocrine syndromes have been reported in association with secondary brain calcification, no specific immunological markers were assessed in our patients. Therefore, autoimmune involvement remains speculative in this series. Infectious, autoimmune, serological, and toxicological evaluations were unremarkable when performed, supporting a secondary metabolic etiology rather than an infectious or toxic cause of intracranial calcification. Likewise, additional hematological parameters, such as ferritin and folate, were also assessed to evaluate concomitant anemia, but were not considered diagnostic markers for intracranial calcification.

Early diagnosis and multidisciplinary management are essential in preventing long‐term neurological and psychiatric complications in Fahr's syndrome. As demonstrated in our case series, collaboration among neurologists, endocrinologists, dermatologists, and psychiatrists allowed for a comprehensive treatment strategy, including seizure control, hormonal correction, dermatologic care, and psychiatric support. This integrative approach facilitated notable clinical improvement, particularly in cognitive function, seizure control, and dermatological symptoms. Early calcium and vitamin D supplementation, as well as hormone replacement therapy, helped normalize biochemical parameters and reduce the progression of neurodegenerative changes, consistent with earlier findings [5, 11, 14]. Prednisolone was initiated primarily for dermatological manifestations and not as disease‐modifying therapy for intracranial calcification.

Recent case reports continue to highlight the diverse clinical spectrum of Fahr's syndrome secondary to hypoparathyroidism. Nisar et al. reported a young adult presenting with seizures and cognitive decline secondary to hypoparathyroidism, with characteristic bilateral intracranial calcifications on CT, highlighting the importance of evaluating calcium‐phosphate metabolism in patients presenting with unexplained neurological manifestations [15]. Like that report, our patients presented with seizures and movement disorders associated with low parathyroid hormone levels and characteristic neuroimaging findings. Our case series further illustrates the coexistence of hypothyroidism and mucocutaneous manifestations in some patients, highlighting the broader spectrum of endocrine abnormalities encountered in secondary Fahr's syndrome. These findings reinforce the importance of early evaluation and a multidisciplinary approach to diagnosis and management [11, 15, 16, 17].

In conclusion, hypoparathyroidism appears to be the primary driver in our case series. While associated anemia, hypothyroidism, and dermatological manifestations were present, further multicenter studies with genetic and immunological evaluation are required to establish a formal link between endocrinological abnormalities and secondary Fahr's syndrome. This series is limited by its size and lack of genetic confirmation, but it underscores the importance of metabolic screening in patients presenting new‐onset seizures and extrapyramidal movements.

## Author Contributions

**Muhammad Husnain Ahmad:** writing – original draft, writing – review and editing, conceptualization. **Haseeb Ahmad:** conceptualization, writing – original draft. **Amna Suhail:** writing – original draft, writing – review and editing. **Ali Gohar:** conceptualization. **Moeez Ahmad Saeed:** writing – original draft, writing – review and editing. **Arham Saif:** writing – original draft, writing – review and editing. **Azka Irfan:** writing – original draft, writing – review and editing, conceptualization. **Ruman Shahzad:** visualization, validation. **Humza Tariq:** writing – original draft, writing – review and editing, visualization.

## Funding

The authors have nothing to report.

## Ethics Statement

The authors have nothing to report.

## Consent

Written informed consent was obtained from the patient based on the journal's policies.

## Conflicts of Interest

The authors declare no conflicts of interest.

## Data Availability

The authors have nothing to report.
